# Supplementary material for: A systematic review of Leptospira in water and soil environments
Source: PLoS One. 2020 Jan 27;15(1):e0227055. doi: 10.1371/journal.pone.0227055 (PMC6984726; doi:10.1371/journal.pone.0227055)
Supplement: Attachment — Submitted filename: Bierque et al response to reviewers comments.docx [file pone.0227055.s002.docx]

**Responses to reviewers comments**

**Reviewer #1:**

This is a timely review of a little understood part of Leptospira spp. transmission and the role contaminated environments may play for the infection of animals and humans. I believe this will be of interest to the field and that it could stimulate much needed research in the area.

We thank Reviewer #1 for this positive appreciation of our work.

**Reviewer #2:**

The manuscript entitled “A systematic review of Leptospira in water and soil environments” bring interesting information from evidence of survival of Leptospira ssp. the environment. The systematic review data are consistent and well presented in the manuscript.

We thank Reviewer #2 for this positive appreciation of our work.

However some minor revisions are needed before the manuscript be considered for publication:

Material and Methods:

Line 85: Is it possible updates the articles until 2019?

This is an interesting suggestion, but would not be a minor change. The process of a systematic review is very tedious and time-consuming. I am afraid that we will be unable to include 2019 in the systematic review. However, I want to draw the attention of Reviewer #2 to the fact that we have included recent literature when relevant, notably in the discussion section. This includes the use of the new phylogeny (subclades P1, P2, S1 and S2, as described in Vincent et al., PLoS NTD 2019, cited as Ref#47), but also the very fascinating study of *Leptospira* in water in Japan using environmental DNA (Sato et al., Sci. Rep. 2019, cited as Ref#126 in the discussion).

Discussion:

- The authors report generally that all pathogenic Leptospira species can survive in the environment. I suggest that some Leptospira spp. that has been found to be in soil or water should be better specifically discussed, considering the species;

This comment definitely makes sense and we thank the reviewer for pointing to this oversimplification. We hesitated to address this issue, but we are happy to do so. As you will see, we now have distinguished 4 known categories in the revision:

- *Leptospira* from the P2 subclade (“intermediates”), not known to have an animal reservoir and probably perfectly adapted to an environmental lifestyle;
- Virulent *Leptospira* spp that survive in the environment and are involved in disease in humans / animals (e.g. *L. interrogans*), the leptospirosis model;
- Virulent *Leptospira* spp that cause disease in humans / animals but are considered unable to survive outside a host (typically *Leptospira borgpetersenii*)
- *Leptospira* spp. from the P1 subclade that are only known from water and soils and probably unable to cause disease in humans / mammals (typically *Leptospira kmetyi*, but also several other species described recently from soils and water).

We hope that the changes made to the MS correctly respond to this comment.

Figure 2 - I suggest deleting Figure 2, it's uninformative;

We disagree with this comment that this figure is uninformative. This figure was built to summarize a hypothetical mechanism originating from the extensive literature review. As mentioned in the figure legend, this figure is also related to Table 2 that describes studies supporting this hypothetical mechanism. Of note, to take into account the comments of Reviewer #3, we have slightly modified the figure and resubmit a revised figure 2. I trust that Reviewer #2 will accept that this figure, together with the Table 2, can be included in the revised MS.

Table 1 - Table 1 shows different Leptospira spp. under various experimental conditions. The table is large and detailed; however, few conclusions were drawn from it.

We acknowledge that Table 1 is a very large table with lots of information. This Table is actually a major output of the literature review and detailed analysis of the articles. Although not systematically cited in the MS (initially only three times, now 5 times in the revision, notably at the start of the paragraphs dealing with *Leptospira* survival in water and in soils), we trust it is an integral component of our article. We kindly ask that this Table be included in the main manuscript, provided the layout can be achieved by typesetting. We trust that moving this Table to Supplementary online material would be detrimental to the article.

**Reviewer #3:**

The manuscript A systematic review of Leptospira in water and soil environments by Bierque et al. is a comprehensive review on a subject that has increasingly been studied in the recent years. Due to the One Health approach, leptospires of human and animal origin have been studied in comparison to environment. Nevertheless, several points remain to be better understood, mainly regarding pathogenic strains.

The manuscript is well written and methodology is adequate.

We thank Reviewer #3 for this positive appreciation of our work.

Nevertheless, my main criticism is that very few associations with the disease on humans and animals were discussed. The context of One Health should be better considered, including animal reservoirs and hosts. Due to its substantial importance nowadays, I strongly suggest that authors explore the One Health framework, including a friendlier look at reservoirs and hosts.

We acknowledge that the focus of our review was actually to collect and analyze all relevant literature about the lifestyle and survival of *Leptospira* spp. in the environment. As a result, our MS probably does not include much about leptospirosis as a disease of humans and mammals. We also acknowledge that leptospirosis as a disease is somehow THE perfect model of a One Health disease. Of note, our systematic review identified a number of case (or outbreak) reports, but the study of environmental contamination of humans or the link between humans / animals / the environment was almost never investigated nor described, as mentioned in our results section. We trust that this weakness of our MS likely reflects the very same weakness of leptospirosis scientific literature. Still, we trust that this comment makes much sense and that an increased consideration of the One Health approach of leptospirosis would hold promising and useful results, so we have tried to include this concept in several occasions in our revised MS.

Besides that, the article presents many speculations and hypotheses, and few certainties.

Again, we acknowledge that there are few certainties, but we trust that this lack of certainties originates in the very same lack of scientific evidence. We initially thought that we could be able to conduct some kind of meta-analysis, which actually proved impossible. The very best we could propose is a hypothetical mechanism of *Leptospira* environmental survival and dispersion upon heavy rainfall, which we have summarized in Figure 2 and Table 2.

Regarding figure 2, I suggest to analyze some issues that are being illustrated and verify if they in fact occur:

We thank Reviewer #3 for pointing to the extreme scenario depicted in our initial Figure 2. We detail below the 3 changes made to our revised Figure 2.

1. When there is no rain, Leptospiras are mostly found in undersoil, not in soil.

Actually and of interest, the paper by Saito and colleagues (cited as Ref #11 in our MS, PMID: 23144130) identified *Leptospira* in a deep soil layer (> 3 cm, with higher water content) during a dry period, then in surface soil on rainy days. We acknowledge that this is to date only limited evidence of vertical migration of *Leptospira* in soil in response to water content.

But in fact, our figure did not aim at distinguishing “undersoil” from “soil”. We have modified Figure 2 to bring *Leptospira* closer to the soil surface. In sediments and river banks, where Leptospires are also abundant (PMID:28241042, cited as Ref #13), the bacteria may be transferred into the water body by passive mobilization by the increased water flow upon rainfall.

2. When there is no rain, no Leptospiras are found in the water.

Our illustration was possibly somewhat excessive in this regard, and the revised Figure 2 shows low number of leptospires during non-rainy periods.

3. When there is rain, no Leptospiras are found in the soil.

Very similarly, we have modified Figure 2 with some leptospires still in the soil during a rainfall event.
